# SpaLLM: a general framework for spatial domain identification with large language models

**DOI:** 10.3389/fbinf.2025.1713975

**Published:** 2026-01-12

**Authors:** Zeyu Zou, Ziheng Duan

**Affiliations:** Department of Computer Science, University of California, Irvine, Irvine, CA, United States

**Keywords:** graph neural networks, large language models, multimodality, spatial domain identification, spatial transcriptomics

## Abstract

Spatial transcriptomics (ST) technologies enable the profiling of gene expression while preserving spatial context, offering unprecedented insights into tissue organization. However, traditional spatial domain identification methods primarily rely on gene expression matrices and spatial coordinates while overlooking the rich biological knowledge encoded in gene functional descriptions. Here, we propose SpaLLM, a general framework that integrates large language model (LLM) embeddings of gene descriptions with conventional spatial transcriptomics analysis. Our approach leverages pre-computed GenePT embeddings from NCBI gene summaries to create biologically-informed gene representations. SpaLLM combines these LLM-derived gene features with cell-gene expression matrices through matrix multiplication, generating enriched cell representations that capture both expression patterns and functional knowledge. These enriched features are then integrated with existing graph-based spatial analysis methods for improved spatial domain identification. Extensive validation on 12 sequencing-based Visium sections and an independent imaging-based osmFISH dataset demonstrate that SpaLLM consistently enhances spatial domain identification. Our modular framework can be seamlessly integrated with existing spatial analysis pipelines, making it broadly applicable to diverse research scenarios.

## 1 Introduction

Spatial transcriptomics (ST) technologies have revolutionized our understanding of tissue architecture by enabling simultaneous measurement of gene expression and spatial location information (Rodriques et al., 2019; Emani et al., 2024; Ruzicka et al., 2024). A fundamental task in ST analysis is spatial domain identification, which aims to partition tissue sections into distinct regions based on similar gene expression patterns and spatial proximity. These spatial domains often correspond to anatomical structures, functional units, or pathological states, making their accurate identification crucial for understanding tissue organization and disease mechanisms (Chen et al., 2022).

Current spatial domain identification methods predominantly follow a graph-based approach, where spots or cells are represented as nodes in a spatial graph, and edges encode spatial proximity relationships (Hu et al., 2021; Zhao et al., 2021; Duan et al., 2024a; Duan et al., 2024b; Duan et al., 2025b). These methods typically employ graph neural networks (GNNs) or graph autoencoders to learn latent representations from cell-by-gene expression matrices and spatial coordinates, followed by clustering algorithms to identify spatial domains (Dong and Zhang, 2022; Long et al., 2023). While effective, these approaches have a fundamental limitation: they treat genes merely as numerical features without leveraging the extensive biological knowledge accumulated about gene functions, pathways, and interactions.

Recent advances in large language models (LLMs) have demonstrated remarkable capabilities in understanding biological text. The GenePT framework has shown that LLM embeddings of gene descriptions from NCBI can effectively capture biological relationships and improve downstream tasks in single-cell analysis (Chen and Zou, 2024). Specifically, GenePT uses pre-computed OpenAI text embeddings on NCBI gene summaries, demonstrating that these embeddings often outperform expression-based methods for various biological tasks.

**FIGURE 1:**
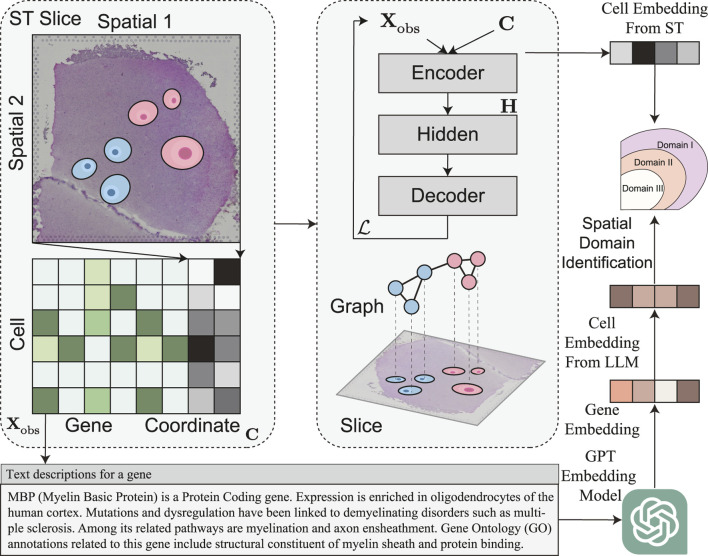
Overview of SpaLLM framework for integrating gene functional knowledge into spatial domain identification. Left: Input spatial transcriptomics data consists of tissue slices with observed gene expression matrix $X_{\text{obs}}\in R^{n\times m}$ and spatial coordinates $C\in R^{n\times 2}$ . Middle: SpaLLM operates through dual complementary pathways: (1) Traditional encoder-decoder architecture processes expression data $X_{\text{obs}}$ and coordinates C through spatial graph neural networks to generate spatially-aware cell embeddings $H_{ST}\in R^{n\times d_{ST}}$ ; (2) Gene functional descriptions from NCBI database (exemplified by MBP gene summary) are encoded using pre-trained GPT embedding models to create gene feature matrix $F\in R^{m\times d_{\mathrm{LLM}}}$ , which is then multiplied with expression matrix to produce functional cell embeddings $H_{\mathrm{LLM}}=X_{\text{obs}}F$ . Right: The two embedding streams—capturing expression patterns with spatial context and gene functional characteristics, respectively—are integrated through weighted combination and fed into clustering algorithms for enhanced spatial domain identification, leveraging both quantitative expression data and qualitative biological knowledge.

Motivated by these observations, we propose SpaLLM, as shown in Figure 1, a general framework that integrates LLM-derived gene functional features with traditional spatial transcriptomics analysis. Our key insight is that gene functional descriptions contain rich biological knowledge that can inform spatial domain identification beyond what expression patterns alone can reveal. By leveraging pre-trained language models to encode gene descriptions from biological databases, we create biologically-informed gene representations that capture functional relationships, pathway memberships, and molecular mechanisms.

The SpaLLM framework introduces a novel approach that enhances traditional spatial transcriptomics analysis by integrating gene functional knowledge. Following the standard encoder-decoder paradigm, we first obtain cell embeddings from ST data using existing graph-based methods. Simultaneously, we derive functional cell embeddings by multiplying the cell-by-gene expression matrix with LLM-derived gene embeddings $\left(H=X_{\text{obs}}F\right)$ , where the gene feature matrix $F\in R^{m\times d}$ encodes biological functions extracted from NCBI descriptions. We then combine these two complementary cell representations—one capturing expression patterns and spatial context, the other encoding functional characteristics—and feed the integrated features into clustering algorithms for improved spatial domain identification.

Our main contributions are as follows:

- We introduce the first systematic framework to integrate LLM embeddings of gene functional descriptions into spatial transcriptomics analysis;
- We demonstrate that incorporating biological knowledge through gene text descriptions significantly improves spatial domain identification accuracy, particularly for low-quality data;
- We provide a modular framework compatible with existing spatial analysis methods, enabling broad adoption across different research pipelines;
- We conduct comprehensive experiments across multiple datasets with varying quality levels, showing consistent 3.8%–400% improvements over state-of-the-art methods.

## 2 Methods

### 2.1 Problem formulation and SpaLLM architecture

We formulate the problem of spatial domain identification as a clustering task on a graph. Let $X\in R^{n\times m}$ be the raw gene expression matrix, where n is the number of spatial spots and m is the number of genes. The spatial coordinates of the spots are given by the matrix $C\in R^{n\times 2}$ . The goal is to learn a mapping f:(X,C)→Z that transforms the input data into a latent feature space $Z\in R^{n\times d_{\mathrm{final}}}$ , where $d_{\mathrm{final}}$ is the final embedding dimension. Subsequently, a clustering algorithm C is applied to Z to identify the spatial domains, i.e., D=C(Z) .

The SpaLLM framework enhances this process by incorporating biological knowledge from gene functional descriptions. We introduce a dual-stream encoding architecture that combines a conventional spatial encoder with a novel LLM-based functional encoder, as illustrated in a figure. The outputs of these two encoders are fused to produce the final enriched embeddings Z , which are then used for clustering.

### 2.2 Gene functional encoding

To capture the biological meaning of genes, we leverage pre-computed embeddings from a large language model trained on a corpus of gene functional descriptions (e.g., NCBI gene summaries). Let $F\in R^{m\times d_{\mathrm{LLM}}}$ be the pre-computed gene feature matrix, where each row $f_{j}\in R^{d_{\mathrm{LLM}}}$ is the vector representation of the j -th gene’s description. This feature matrix serves as a look-up table for the functional representation of each gene. The functional encoder transforms the gene expression data into a functional feature space. We define the functional spot embeddings $H_{\mathrm{LLM}}\in R^{n\times d_{\mathrm{LLM}}}$ as:

$$H_{\mathrm{LLM}}=E_{\mathrm{func}}\left(X\right)=XF$$

Here, $E_{\mathrm{func}}$ is the functional encoder. This operation implicitly weighs the contribution of each functional dimension based on the expression level of corresponding genes in each spot.

### 2.3 Spatial encoder

The spatial encoder captures both gene expression patterns and spatial context. We construct a spatial graph G=(V,E) where the set of vertices V corresponds to the n spots. The edge weights are defined by the adjacency matrix A , where $A_{ij}$ represents the spatial proximity between spot i and spot j . A common choice for computing A is a Gaussian kernel on the spatial coordinates:

$$A_{ij}=\exp\left(-\frac{\|c_{i}-c_{j}{\|}^{2}}{2\sigma^{2}}\right)\;\text{if }\|c_{i}-c_{j}\|\leq \tau ,\text{ otherwise }0$$

Here, σ and τ are hyperparameters.

The spatial encoder, $E_{\mathrm{spat}}$ , is a Graph Neural Network (GNN) that learns spatially-aware spot embeddings $H_{ST}\in R^{n\times d_{ST}}$ by propagating and aggregating features over the graph. The input to the GNN is the normalized gene expression matrix, $X_{\mathrm{norm}}$ :

$$H_{ST}=E_{\mathrm{spat}}\left(X_{\mathrm{norm}},A\right)$$

The GNN layers are typically defined by a message passing scheme. For a multi-layer GNN, the update rule for the l -th layer is:

$$H^{\left(l+1\right)}=\sigma \left(\hat{A}H^{\left(l\right)}W^{\left(l\right)}\right)$$

where $H^{\left(0\right)}=X_{\mathrm{norm}}$ , $\hat{A}$ is the normalized adjacency matrix, $W^{\left(l\right)}$ is a trainable weight matrix, and σ(⋅) is a non-linear activation function.

### 2.4 Feature fusion and clustering

The embeddings from the two encoders, $H_{ST}\in R^{n\times d_{ST}}$ and $H_{\mathrm{LLM}}\in R^{n\times d_{\mathrm{LLM}}}$ , may have different dimensions $\left(d_{ST}\neq d_{\mathrm{LLM}}\right)$ . To ensure feature alignment before fusion, we first perform Principal Component Analysis (PCA) on the LLM embeddings to project them into the same dimension as the spatial embeddings. This yields the dimension-reduced functional embeddings, $H_{\mathrm{LLM}}^{\mathrm{PCA}}\in R^{n\times d_{ST}}$ .

The final spot representation $Z\in R^{n\times d_{ST}}$ is then obtained by a weighted combination of the spatial and dimension-reduced functional embeddings:

$$Z=F\left(H_{ST},H_{\mathrm{LLM}}^{\mathrm{PCA}}\right)=\alpha \cdot H_{ST}+\beta \cdot H_{\mathrm{LLM}}^{\mathrm{PCA}}$$

The hyperparameters α and β control the contribution of each embedding stream. The combined embeddings Z capture both spatial proximity/expression patterns and biological functional knowledge.

Finally, the spatial domains are identified by applying a clustering algorithm, such as K-means, Louvain, or mclust, to the fused feature matrix Z .

## 3 Experimental setup

### 3.1 Datasets and data quality simulation

We evaluate SpaLLM on the human dorsolateral prefrontal cortex (DLPFC) spatial transcriptomics datasets from Maynard et al. (2021), which consist of 12 tissue sections with manually annotated spatial domains. To assess the robustness of our framework against varying data quality, we adopt a systematic simulation strategy where data quality is reduced by introducing sparsity Duan et al. (2025a). The original, unaltered data serves as a baseline for comparison. We create four simulated quality levels by randomly masking a percentage of the non-zero gene expression values: Q1 (50% masked), Q2 (75% masked), Q3 (87.5% masked), and Q4 (93.75% masked). This process generates a comprehensive testbed of 48 simulated datasets (12 original sections × 4 quality levels), enabling a robust evaluation of SpaLLM’s performance under different conditions. The characteristics of these datasets are summarized in Table 1.

**TABLE 1:** Summary of datasets and quality simulation.

Slice ID	Dimensions (cells × genes)	Original density (%)	Quality level density (%)			
			Q1	Q2	Q3	Q4
151507	(4221, 3000)	12.75	6.38	3.19	1.60	0.80
151508	(4381, 3000)	11.96	5.98	2.99	1.50	0.75
151509	(4788, 3000)	12.92	6.46	3.23	1.62	0.81
151510	(4595, 3000)	12.88	6.44	3.22	1.61	0.81
151669	(3636, 3000)	12.71	6.36	3.18	1.59	0.80
151670	(3484, 3000)	12.72	6.36	3.18	1.59	0.80
151671	(4093, 3000)	13.70	6.85	3.43	1.72	0.86
151672	(3888, 3000)	13.41	6.70	3.35	1.68	0.84
151673	(3611, 3000)	15.18	7.59	3.80	1.90	0.95
151674	(3635, 3000)	16.81	8.41	4.20	2.10	1.05
151675	(3566, 3000)	13.60	6.80	3.40	1.70	0.85
151676	(3431, 3000)	14.36	7.18	3.59	1.80	0.90

To validate cross-platform generalizability, we incorporated the osmFISH dataset (Codeluppi et al., 2018) of the mouse somatosensory cortex. This dataset utilizes imaging-based technology, which provides a higher spatial resolution but fewer genes compared to the sequencing-based Visium platform, offering a complementary modality for testing.

### 3.2 Implementation details

#### 3.2.1 GenePT feature configuration

We use the pre-computed GenePT embeddings (Chen and Zou, 2024) with the following specifications:

- Embedding model: OpenAI *text-embedding-ada-002.*
- Feature dimension: d=1536 .
- Gene coverage: 33,000+ genes with NCBI annotations.

#### 3.2.2 Model hyperparameters

We adopt four representative baselines: SpaceFlow (Ren et al., 2022), STAGATE (Dong and Zhang, 2022), GraphST (Long et al., 2023), and stCluster (Wang et al., 2024). The hyperparameters for each method are set following the configurations recommended in their original papers. For SpaLLM integration, we set the weighting parameters α=0.5 and β=0.5 as default values to achieve balanced integration between spatial expression features and functional embeddings.

### 3.3 Evaluation metrics

We evaluate spatial domain identification using the Adjusted Rand Index (ARI), a robust metric for measuring the similarity between a predicted clustering and the ground truth. The ARI corrects for chance agreements and has a value of 1.0 for perfect clustering and 0 for random assignments. The formula for ARI is defined as:

$$ARI=\frac{RI-E\left[RI\right]}{max\left(RI\right)-E\left[RI\right]}$$

Where RI is the Rand Index, E[RI] is the expected Rand Index for a random partition, and max(RI) is the maximum possible Rand Index.

## 4 Results

### 4.1 SpaLLM demonstrates consistent improvements across quality levels

We evaluated SpaLLM’s performance on spatial domain identification across 12 real-world and 48 simulated datasets with varying quality levels (Q0-Q4, where Q0 represents the original highest quality datasets and Q4 the lowest). Tables 2–4 present comprehensive results comparing four baseline methods (SpaceFlow, STAGATE, GraphST, and stCluster) with their SpaLLM-enhanced versions across three different donor samples. Results are averaged across ten runs.

**TABLE 2:** Spatial domain identification performance (ARI) on Donor 1 datasets. The best performance for each quality level and dataset is bolded.

Method	Quality	Donor 1			
		151507	151508	151509	151510
SpaceFlow	Q0	$0.41_{\pm 0.02}$	$0.38_{\pm 0.03}$	$0.34_{\pm 0.04}$	$0.36_{\pm 0.02}$
	Q1	$0.39_{\pm 0.02}$	$0.36_{\pm 0.04}$	$0.32_{\pm 0.04}$	$0.34_{\pm 0.02}$
	Q2	$0.38_{\pm 0.04}$	$0.30_{\pm 0.04}$	$0.31_{\pm 0.04}$	$0.32_{\pm 0.07}$
	Q3	$0.17_{\pm 0.03}$	$0.16_{\pm 0.02}$	$0.25_{\pm 0.04}$	$0.18_{\pm 0.02}$
	Q4	$0.07_{\pm 0.01}$	$0.10_{\pm 0.03}$	$0.05_{\pm 0.07}$	$0.06_{\pm 0.03}$
SpaceFlow + SpaLLM	Q0	$0.43_{\pm 0.02}$	$0.40_{\pm 0.03}$	$0.36_{\pm 0.04}$	$0.38_{\pm 0.02}$
	Q1	$0.42_{\pm 0.02}$	$0.39_{\pm 0.04}$	$0.35_{\pm 0.04}$	$0.37_{\pm 0.02}$
	Q2	$0.41_{\pm 0.04}$	$0.33_{\pm 0.04}$	$0.34_{\pm 0.04}$	$0.35_{\pm 0.07}$
	Q3	$0.20_{\pm 0.03}$	$0.19_{\pm 0.02}$	$0.28_{\pm 0.04}$	$0.21_{\pm 0.02}$
	Q4	$0.10_{\pm 0.01}$	$0.13_{\pm 0.03}$	$0.08_{\pm 0.07}$	$0.09_{\pm 0.03}$
STAGATE	Q0	$0.55_{\pm 0.02}$	$0.49_{\pm 0.05}$	$0.47_{\pm 0.07}$	$0.42_{\pm 0.04}$
	Q1	$0.53_{\pm 0.02}$	$0.47_{\pm 0.05}$	$0.45_{\pm 0.07}$	$0.40_{\pm 0.04}$
	Q2	$0.22_{\pm 0.05}$	$0.25_{\pm 0.09}$	$0.31_{\pm 0.02}$	$0.30_{\pm 0.03}$
	Q3	$0.12_{\pm 0.07}$	$0.06_{\pm 0.08}$	$0.10_{\pm 0.11}$	$0.15_{\pm 0.11}$
	Q4	$0.02_{\pm 0.00}$	$0.01_{\pm 0.01}$	$0.02_{\pm 0.01}$	$0.02_{\pm 0.00}$
STAGATE + SpaLLM	Q0	$0.58_{\pm 0.02}$	$0.52_{\pm 0.05}$	$0.50_{\pm 0.07}$	$0.45_{\pm 0.04}$
	Q1	$0.56_{\pm 0.02}$	$0.50_{\pm 0.05}$	$0.48_{\pm 0.07}$	$0.43_{\pm 0.04}$
	Q2	$0.26_{\pm 0.05}$	$0.29_{\pm 0.09}$	$0.35_{\pm 0.02}$	$0.34_{\pm 0.03}$
	Q3	$0.16_{\pm 0.07}$	$0.10_{\pm 0.08}$	$0.14_{\pm 0.11}$	$0.19_{\pm 0.11}$
	Q4	$0.06_{\pm 0.00}$	$0.05_{\pm 0.01}$	$0.06_{\pm 0.01}$	$0.06_{\pm 0.00}$
GraphST	Q0	$0.46_{\pm 0.08}$	$0.43_{\pm 0.07}$	$0.44_{\pm 0.06}$	$0.46_{\pm 0.05}$
	Q1	$0.44_{\pm 0.08}$	$0.41_{\pm 0.07}$	$0.42_{\pm 0.06}$	$0.44_{\pm 0.05}$
	Q2	$0.21_{\pm 0.07}$	$0.22_{\pm 0.05}$	$0.38_{\pm 0.13}$	$0.39_{\pm 0.03}$
	Q3	$0.02_{\pm 0.06}$	$0.03_{\pm 0.05}$	$0.03_{\pm 0.05}$	$0.01_{\pm 0.01}$
	Q4	$0.03_{\pm 0.05}$	$0.01_{\pm 0.02}$	$0.01_{\pm 0.03}$	$0.01_{\pm 0.01}$
GraphST + SpaLLM	Q0	$0.50_{\pm 0.08}$	$0.47_{\pm 0.07}$	$0.48_{\pm 0.06}$	$0.50_{\pm 0.05}$
	Q1	$0.48_{\pm 0.08}$	$0.45_{\pm 0.07}$	$0.46_{\pm 0.06}$	$0.48_{\pm 0.05}$
	Q2	$0.25_{\pm 0.07}$	$0.26_{\pm 0.05}$	$0.42_{\pm 0.13}$	$0.43_{\pm 0.03}$
	Q3	$0.06_{\pm 0.06}$	$0.07_{\pm 0.05}$	$0.07_{\pm 0.05}$	$0.05_{\pm 0.01}$
	Q4	$0.07_{\pm 0.05}$	$0.05_{\pm 0.02}$	$0.05_{\pm 0.03}$	$0.05_{\pm 0.01}$
stCluster	Q0	$0.46_{\pm 0.02}$	$0.37_{\pm 0.02}$	$0.43_{\pm 0.02}$	$0.42_{\pm 0.02}$
	Q1	$0.44_{\pm 0.02}$	$0.35_{\pm 0.02}$	$0.41_{\pm 0.02}$	$0.40_{\pm 0.02}$
	Q2	$0.41_{\pm 0.03}$	$0.29_{\pm 0.01}$	$0.36_{\pm 0.05}$	$0.35_{\pm 0.05}$
	Q3	$0.21_{\pm 0.01}$	$0.15_{\pm 0.02}$	$0.28_{\pm 0.06}$	$0.28_{\pm 0.06}$
	Q4	$0.14_{\pm 0.07}$	$0.12_{\pm 0.00}$	$0.16_{\pm 0.09}$	$0.19_{\pm 0.02}$
stCluster + SpaLLM	Q0	$0.49_{\pm 0.02}$	$0.40_{\pm 0.02}$	$0.46_{\pm 0.02}$	$0.45_{\pm 0.02}$
	Q1	$0.47_{\pm 0.02}$	$0.38_{\pm 0.02}$	$0.44_{\pm 0.02}$	$0.43_{\pm 0.02}$
	Q2	$0.44_{\pm 0.03}$	$0.32_{\pm 0.01}$	$0.39_{\pm 0.05}$	$0.38_{\pm 0.05}$
	Q3	$0.24_{\pm 0.01}$	$0.18_{\pm 0.02}$	$0.31_{\pm 0.06}$	$0.31_{\pm 0.06}$
	Q4	$0.17_{\pm 0.07}$	$0.15_{\pm 0.00}$	$0.19_{\pm 0.09}$	$0.22_{\pm 0.02}$

Bold values indicate the best performance (highest ARI) for each dataset and quality level.

**TABLE 3:** Spatial domain identification performance (ARI) on Donor 2 datasets. The best performance for each quality level and dataset is bolded.

Method	Quality	Donor 2			
		151669	151670	151671	151672
SpaceFlow	Q0	$0.33_{\pm 0.05}$	$0.34_{\pm 0.04}$	$0.53_{\pm 0.03}$	$0.50_{\pm 0.05}$
	Q1	$0.31_{\pm 0.06}$	$0.32_{\pm 0.04}$	$0.51_{\pm 0.03}$	$0.48_{\pm 0.06}$
	Q2	$0.26_{\pm 0.09}$	$0.19_{\pm 0.04}$	$0.33_{\pm 0.04}$	$0.41_{\pm 0.04}$
	Q3	$0.21_{\pm 0.07}$	$0.13_{\pm 0.02}$	$0.20_{\pm 0.10}$	$0.11_{\pm 0.04}$
	Q4	$0.06_{\pm 0.04}$	$0.08_{\pm 0.01}$	$0.08_{\pm 0.02}$	$0.11_{\pm 0.03}$
SpaceFlow + SpaLLM	Q0	$0.35_{\pm 0.05}$	$0.36_{\pm 0.04}$	$0.55_{\pm 0.03}$	$0.52_{\pm 0.05}$
	Q1	$0.34_{\pm 0.06}$	$0.35_{\pm 0.04}$	$0.54_{\pm 0.03}$	$0.51_{\pm 0.06}$
	Q2	$0.29_{\pm 0.09}$	$0.22_{\pm 0.04}$	$0.36_{\pm 0.04}$	$0.44_{\pm 0.04}$
	Q3	$0.24_{\pm 0.07}$	$0.16_{\pm 0.02}$	$0.23_{\pm 0.10}$	$0.14_{\pm 0.04}$
	Q4	$0.09_{\pm 0.04}$	$0.11_{\pm 0.01}$	$0.11_{\pm 0.02}$	$0.14_{\pm 0.03}$
STAGATE	Q0	$0.41_{\pm 0.08}$	$0.34_{\pm 0.08}$	$0.57_{\pm 0.04}$	$0.55_{\pm 0.11}$
	Q1	$0.39_{\pm 0.08}$	$0.32_{\pm 0.08}$	$0.55_{\pm 0.04}$	$0.53_{\pm 0.11}$
	Q2	$0.05_{\pm 0.11}$	$0.30_{\pm 0.22}$	$0.25_{\pm 0.17}$	$0.36_{\pm 0.17}$
	Q3	$0.02_{\pm 0.02}$	$0.02_{\pm 0.03}$	$0.10_{\pm 0.04}$	$0.11_{\pm 0.02}$
	Q4	$0.01_{\pm 0.01}$	$0.01_{\pm 0.01}$	$0.02_{\pm 0.01}$	$0.02_{\pm 0.02}$
STAGATE+SpaLLM	Q0	$0.44_{\pm 0.08}$	$0.37_{\pm 0.08}$	$0.60_{\pm 0.04}$	$0.58_{\pm 0.11}$
	Q1	$0.42_{\pm 0.08}$	$0.35_{\pm 0.08}$	$0.58_{\pm 0.04}$	$0.56_{\pm 0.11}$
	Q2	$0.08_{\pm 0.11}$	$0.34_{\pm 0.22}$	$0.29_{\pm 0.17}$	$0.40_{\pm 0.17}$
	Q3	$0.06_{\pm 0.02}$	$0.06_{\pm 0.03}$	$0.14_{\pm 0.04}$	$0.15_{\pm 0.02}$
	Q4	$0.05_{\pm 0.01}$	$0.05_{\pm 0.01}$	$0.06_{\pm 0.01}$	$0.06_{\pm 0.02}$
GraphST	Q0	$0.49_{\pm 0.21}$	$0.43_{\pm 0.09}$	$0.63_{\pm 0.10}$	$0.65_{\pm 0.07}$
	Q1	$0.47_{\pm 0.21}$	$0.41_{\pm 0.09}$	$0.61_{\pm 0.10}$	$0.63_{\pm 0.07}$
	Q2	$0.01_{\pm 0.08}$	$0.07_{\pm 0.12}$	$0.17_{\pm 0.04}$	$0.13_{\pm 0.01}$
	Q3	$0.01_{\pm 0.00}$	$0.01_{\pm 0.01}$	$0.01_{\pm 0.02}$	$0.06_{\pm 0.06}$
	Q4	$0.01_{\pm 0.00}$	$0.01_{\pm 0.00}$	$0.01_{\pm 0.02}$	$0.01_{\pm 0.01}$
GraphST + SpaLLM	Q0	$0.53_{\pm 0.21}$	$0.47_{\pm 0.09}$	$0.67_{\pm 0.10}$	$0.69_{\pm 0.07}$
	Q1	$0.51_{\pm 0.21}$	$0.45_{\pm 0.09}$	$0.65_{\pm 0.10}$	$0.67_{\pm 0.07}$
	Q2	$0.05_{\pm 0.08}$	$0.11_{\pm 0.12}$	$0.21_{\pm 0.04}$	$0.17_{\pm 0.01}$
	Q3	$0.05_{\pm 0.00}$	$0.05_{\pm 0.01}$	$0.05_{\pm 0.02}$	$0.10_{\pm 0.06}$
	Q4	$0.05_{\pm 0.00}$	$0.05_{\pm 0.00}$	$0.05_{\pm 0.02}$	$0.05_{\pm 0.01}$
stCluster	Q0	$0.38_{\pm 0.09}$	$0.37_{\pm 0.05}$	$0.55_{\pm 0.03}$	$0.64_{\pm 0.10}$
	Q1	$0.36_{\pm 0.09}$	$0.35_{\pm 0.05}$	$0.53_{\pm 0.03}$	$0.62_{\pm 0.10}$
	Q2	$0.25_{\pm 0.09}$	$0.21_{\pm 0.02}$	$0.44_{\pm 0.02}$	$0.34_{\pm 0.11}$
	Q3	$0.24_{\pm 0.08}$	$0.14_{\pm 0.07}$	$0.28_{\pm 0.07}$	$0.24_{\pm 0.04}$
	Q4	$0.14_{\pm 0.05}$	$0.11_{\pm 0.08}$	$0.09_{\pm 0.02}$	$0.06_{\pm 0.02}$
stCluster + SpaLLM	Q0	$0.41_{\pm 0.09}$	$0.40_{\pm 0.05}$	$0.58_{\pm 0.03}$	$0.67_{\pm 0.10}$
	Q1	$0.39_{\pm 0.09}$	$0.38_{\pm 0.05}$	$0.56_{\pm 0.03}$	$0.65_{\pm 0.10}$
	Q2	$0.28_{\pm 0.09}$	$0.24_{\pm 0.02}$	$0.47_{\pm 0.02}$	$0.37_{\pm 0.11}$
	Q3	$0.27_{\pm 0.08}$	$0.17_{\pm 0.07}$	$0.31_{\pm 0.07}$	$0.27_{\pm 0.04}$
	Q4	$0.17_{\pm 0.05}$	$0.14_{\pm 0.08}$	$0.12_{\pm 0.02}$	$0.09_{\pm 0.02}$

Bold values indicate the best performance (highest ARI) for each dataset and quality level.

**TABLE 4:** Spatial domain identification performance (ARI) on Donor 3 datasets. The best performance for each quality level and dataset is bolded.

Method	Quality	Donor 3			
		151673	151674	151675	151676
SpaceFlow	Q0	$0.39_{\pm 0.04}$	$0.34_{\pm 0.02}$	$0.41_{\pm 0.05}$	$0.38_{\pm 0.03}$
	Q1	$0.37_{\pm 0.04}$	$0.32_{\pm 0.02}$	$0.39_{\pm 0.06}$	$0.36_{\pm 0.03}$
	Q2	$0.31_{\pm 0.02}$	$0.28_{\pm 0.01}$	$0.33_{\pm 0.01}$	$0.25_{\pm 0.03}$
	Q3	$0.27_{\pm 0.02}$	$0.26_{\pm 0.03}$	$0.21_{\pm 0.04}$	$0.27_{\pm 0.01}$
	Q4	$0.21_{\pm 0.01}$	$0.21_{\pm 0.02}$	$0.20_{\pm 0.02}$	$0.20_{\pm 0.01}$
SpaceFlow + SpaLLM	Q0	$0.41_{\pm 0.04}$	$0.36_{\pm 0.02}$	$0.43_{\pm 0.05}$	$0.40_{\pm 0.03}$
	Q1	$0.40_{\pm 0.04}$	$0.35_{\pm 0.02}$	$0.42_{\pm 0.06}$	$0.39_{\pm 0.03}$
	Q2	$0.34_{\pm 0.02}$	$0.31_{\pm 0.01}$	$0.36_{\pm 0.01}$	$0.28_{\pm 0.03}$
	Q3	$0.30_{\pm 0.02}$	$0.29_{\pm 0.03}$	$0.24_{\pm 0.04}$	$0.30_{\pm 0.01}$
	Q4	$0.24_{\pm 0.01}$	$0.24_{\pm 0.02}$	$0.23_{\pm 0.02}$	$0.23_{\pm 0.01}$
STAGATE	Q0	$0.57_{\pm 0.04}$	$0.49_{\pm 0.05}$	$0.44_{\pm 0.02}$	$0.53_{\pm 0.09}$
	Q1	$0.55_{\pm 0.04}$	$0.47_{\pm 0.05}$	$0.42_{\pm 0.02}$	$0.51_{\pm 0.09}$
	Q2	$0.36_{\pm 0.04}$	$0.28_{\pm 0.02}$	$0.31_{\pm 0.05}$	$0.33_{\pm 0.02}$
	Q3	$0.16_{\pm 0.03}$	$0.16_{\pm 0.03}$	$0.12_{\pm 0.01}$	$0.15_{\pm 0.03}$
	Q4	$0.03_{\pm 0.01}$	$0.01_{\pm 0.00}$	$0.02_{\pm 0.00}$	$0.01_{\pm 0.01}$
STAGATE + SpaLLM	Q0	$0.60_{\pm 0.04}$	$0.52_{\pm 0.05}$	$0.47_{\pm 0.02}$	$0.56_{\pm 0.09}$
	Q1	$0.58_{\pm 0.04}$	$0.50_{\pm 0.05}$	$0.45_{\pm 0.02}$	$0.54_{\pm 0.09}$
	Q2	$0.40_{\pm 0.04}$	$0.32_{\pm 0.02}$	$0.35_{\pm 0.05}$	$0.37_{\pm 0.02}$
	Q3	$0.20_{\pm 0.03}$	$0.20_{\pm 0.03}$	$0.16_{\pm 0.01}$	$0.19_{\pm 0.03}$
	Q4	$0.07_{\pm 0.01}$	$0.05_{\pm 0.00}$	$0.06_{\pm 0.00}$	$0.05_{\pm 0.01}$
GraphST	Q0	$0.47_{\pm 0.04}$	$0.42_{\pm 0.05}$	$0.33_{\pm 0.10}$	$0.31_{\pm 0.07}$
	Q1	$0.45_{\pm 0.04}$	$0.40_{\pm 0.05}$	$0.31_{\pm 0.10}$	$0.29_{\pm 0.07}$
	Q2	$0.15_{\pm 0.03}$	$0.17_{\pm 0.02}$	$0.14_{\pm 0.04}$	$0.15_{\pm 0.12}$
	Q3	$0.12_{\pm 0.04}$	$0.12_{\pm 0.05}$	$0.15_{\pm 0.03}$	$0.11_{\pm 0.05}$
	Q4	$0.13_{\pm 0.01}$	$0.01_{\pm 0.01}$	$0.08_{\pm 0.05}$	$0.01_{\pm 0.01}$
GraphST + SpaLLM	Q0	$0.51_{\pm 0.04}$	$0.46_{\pm 0.05}$	$0.37_{\pm 0.10}$	$0.35_{\pm 0.07}$
	Q1	$0.49_{\pm 0.04}$	$0.44_{\pm 0.05}$	$0.35_{\pm 0.10}$	$0.33_{\pm 0.07}$
	Q2	$0.19_{\pm 0.03}$	$0.21_{\pm 0.02}$	$0.18_{\pm 0.04}$	$0.19_{\pm 0.12}$
	Q3	$0.16_{\pm 0.04}$	$0.16_{\pm 0.05}$	$0.19_{\pm 0.03}$	$0.15_{\pm 0.05}$
	Q4	$0.17_{\pm 0.01}$	$0.05_{\pm 0.01}$	$0.12_{\pm 0.05}$	$0.05_{\pm 0.01}$
stCluster	Q0	$0.50_{\pm 0.04}$	$0.48_{\pm 0.05}$	$0.37_{\pm 0.03}$	$0.43_{\pm 0.08}$
	Q1	$0.48_{\pm 0.04}$	$0.46_{\pm 0.05}$	$0.35_{\pm 0.03}$	$0.41_{\pm 0.08}$
	Q2	$0.44_{\pm 0.03}$	$0.38_{\pm 0.02}$	$0.32_{\pm 0.06}$	$0.28_{\pm 0.01}$
	Q3	$0.26_{\pm 0.06}$	$0.32_{\pm 0.03}$	$0.25_{\pm 0.01}$	$0.24_{\pm 0.01}$
	Q4	$0.18_{\pm 0.04}$	$0.23_{\pm 0.01}$	$0.19_{\pm 0.03}$	$0.19_{\pm 0.01}$
stCluster + SpaLLM	Q0	$0.53_{\pm 0.04}$	$0.51_{\pm 0.05}$	$0.40_{\pm 0.03}$	$0.46_{\pm 0.08}$
	Q1	$0.51_{\pm 0.04}$	$0.49_{\pm 0.05}$	$0.38_{\pm 0.03}$	$0.44_{\pm 0.08}$
	Q2	$0.47_{\pm 0.03}$	$0.41_{\pm 0.02}$	$0.35_{\pm 0.06}$	$0.31_{\pm 0.01}$
	Q3	$0.29_{\pm 0.06}$	$0.35_{\pm 0.03}$	$0.28_{\pm 0.01}$	$0.27_{\pm 0.01}$
	Q4	$0.21_{\pm 0.04}$	$0.26_{\pm 0.01}$	$0.22_{\pm 0.03}$	$0.22_{\pm 0.01}$

Bold values indicate the best performance (highest ARI) for each dataset and quality level.

The integration of SpaLLM with baseline methods shows consistent improvements across all quality levels and datasets. Notably, the performance gains become more pronounced as data quality decreases, highlighting SpaLLM’s robustness in challenging scenarios where traditional methods struggle.

Across all three donor samples, SpaceFlow integration with SpaLLM achieved modest but consistent improvements ranging from 3.8% to 9.4% in high-quality datasets (Q0, Q1) to more substantial gains of 11.1%–60.0% in degraded datasets (Q3, Q4). For example, in Donor 1 dataset 151509, SpaceFlow improved from 0.25 to 0.28 (12% gain) at Q3 level, while in dataset 151508, Q4 performance increased from 0.10 to 0.13 (30% gain).

STAGATE integration with SpaLLM demonstrated the most substantial improvements among all tested methods. In high-quality scenarios (Q0, Q1), improvements ranged from 5.3% to 9.4%, with notable examples including Donor 1 dataset 151507 improving from 0.55 to 0.58 (5.5% gain) at Q0. However, the most dramatic gains occurred in degraded data scenarios, with Q3 and Q4 improvements reaching 25%–400%. For instance, in the Donor 2 dataset 151670, Q4 performance surged from 0.01 to 0.05 (400% improvement), and in the Donor 1 dataset 151507, Q3 performance increased from 0.12 to 0.16 (33% gain).

GraphST showed significant benefits from SpaLLM integration, with improvements ranging from 6.2% to 13.8% in high-quality datasets to remarkable gains of up to 400% in the most challenging scenarios. In Donor 2, GraphST + SpaLLM achieved the highest overall performance in Q0 and Q1 levels, with values reaching 0.69 and 0.67, respectively, for dataset 151672. The most striking improvements were observed in Q4 scenarios, where performance increased from as low as 0.01 to 0.05 (400% improvement).

stCluster integration yielded improvements across all quality levels. High-quality datasets (Q0, Q1) showed improvements ranging from 4.7% to 8.6%, while degraded scenarios (Q3, Q4) demonstrated gains of 10.0%–50.0%. Notably, stCluster + SpaLLM achieved several best performances in Q2–Q4 categories, such as 0.44 (Q2) and 0.24 (Q3) in the Donor 1 dataset 151507.

### 4.2 Ablation studies: synergistic effects of LLM priors and integration strategies

To dissect the specific contributions of the individual components within the SpaLLM framework, we performed comprehensive ablation studies focusing on the feature integration strategy and the choice of the LLM embedding model. We utilized the STAGATE baseline on both the DLPFC (151507) and osmFISH datasets as representative cases.

As quantified in Table 5, we compared four distinct integration strategies: (1) *Expression only* (standard pipeline), (2) *Functional only* (LLM knowledge only), (3) *Simple concatenation*, and (4) *Weighted fusion* (SpaLLM). The results demonstrate a “synergistic effect.” While expression features are dominant in high-quality data (Q0), they suffer from a catastrophic performance collapse as sparsity increases; for example, the ARI on DLPFC drops from 0.55 (Q0) to a mere 0.02 (Q4). In contrast, the *Functional only* approach maintains remarkable stability (e.g., maintaining an ARI of 0.132 on osmFISH even at Q4), indicating that biological priors act as a vital regularizer when the transcriptomic signal is severely degraded.

**TABLE 5:** Comprehensive ablation study results (ARI) across DLPFC and osmFISH datasets using STAGATE.

Dataset	Strategy/Model	Q0	Q1	Q2	Q3	Q4
DLPFC	Expression only	0.55	0.53	0.22	0.12	0.02
	Functional only	0.54	0.53	0.24	0.13	0.02
	Concatenation	0.56	0.54	0.24	0.14	0.04
	Weighted (ada)	**0.58**	**0.56**	**0.26**	0.16	0.06
	Weighted (large)	**0.58**	0.55	0.25	**0.18**	**0.08**
osmFISH	Expression only	0.397	0.372	0.305	0.182	0.085
	Functional only	0.402	0.374	0.331	0.205	0.132
	Concatenation	0.411	0.389	0.357	0.215	0.142
	Weighted (ada)	0.420	0.401	0.358	0.238	0.168
	Weighted (large)	**0.421**	**0.403**	**0.361**	**0.246**	**0.175**

Bold values indicate the best performance (highest ARI) for each dataset and quality level.

Furthermore, we evaluated two OpenAI embedding models: *text-embedding-ada-002* (ada) and *text-embedding-3-large* (large). Our analysis shows that while *simple concatenation* only yields marginal gains, our *Weighted fusion* strategy achieves the best overall performance. Notably, the *large* model variant exhibits superior robustness in the most challenging scenarios (Q3–Q4), providing the highest ARI across both datasets. However, *ada* offers a comparable balance with lower computational overhead, which we selected as the default configuration for general efficiency.

### 4.3 Cross-platform generalizability: validation on osmFISH

To ensure that SpaLLM is technology-agnostic, we extended our evaluation to the osmFISH dataset (mouse somatosensory cortex). Unlike sequencing-based platforms, osmFISH is an imaging-based technology with a high spatial resolution but a specific gene panel (33 marker genes).

We applied SpaLLM to four representative baselines: SpaceFlow, STAGATE, GraphST, and stCluster. As summarized in Table 6, SpaLLM consistently improved the ARI across all quality levels for every baseline. For instance, GraphST + SpaLLM achieved the highest ARI of 0.52 at Q0 (compared to 0.48 for base GraphST) and maintained a significant lead even at Q4 (0.18 vs. 0.09). These results, accompanied by lower performance variance (standard deviations), prove that integrating LLM-derived knowledge provides a universal enhancement for spatial domain identification that is independent of the underlying experimental modality or algorithmic architecture.

**TABLE 6:** Full performance comparison (ARI) on the osmFISH dataset across different data quality levels.

Method	Q0	Q1	Q2	Q3	Q4
SpaceFlow	$0.46_{\pm 0.03}$	$0.43_{\pm 0.03}$	$0.37_{\pm 0.04}$	$0.29_{\pm 0.03}$	$0.21_{\pm 0.02}$
SpaceFlow + SpaLLM	$0.49_{\pm 0.03}$	$0.47_{\pm 0.03}$	$0.41_{\pm 0.04}$	$0.34_{\pm 0.03}$	$0.27_{\pm 0.02}$
STAGATE	$0.39_{\pm 0.04}$	$0.36_{\pm 0.04}$	$0.29_{\pm 0.03}$	$0.18_{\pm 0.02}$	$0.08_{\pm 0.01}$
STAGATE + SpaLLM	$0.43_{\pm 0.04}$	$0.40_{\pm 0.04}$	$0.33_{\pm 0.03}$	$0.23_{\pm 0.02}$	$0.14_{\pm 0.01}$
GraphST	$0.48_{\pm 0.05}$	$0.45_{\pm 0.05}$	$0.31_{\pm 0.04}$	$0.17_{\pm 0.03}$	$0.09_{\pm 0.02}$
GraphST + SpaLLM	$0.52_{\pm 0.05}$	$0.49_{\pm 0.05}$	$0.37_{\pm 0.04}$	$0.25_{\pm 0.03}$	$0.18_{\pm 0.02}$
stCluster	$0.44_{\pm 0.03}$	$0.42_{\pm 0.03}$	$0.36_{\pm 0.04}$	$0.28_{\pm 0.03}$	$0.22_{\pm 0.02}$
stCluster + SpaLLM	$0.47_{\pm 0.03}$	$0.45_{\pm 0.03}$	$0.40_{\pm 0.04}$	$0.33_{\pm 0.03}$	$0.28_{\pm 0.02}$

Bold values indicate the best performance (highest ARI) for each dataset and quality level.

### 4.4 Practical guidance for low-throughput and small-sample regions

To provide concrete guidance for practitioners working with limited tissue sections, we analyzed the performance of SpaLLM on small spatial subregions. We randomly extracted 10 contiguous subregions, each consisting of only 1,000 cells, from the osmFISH tissue.

As summarized in Table 7, the relative performance improvement introduced by SpaLLM is even more pronounced in these small-sample scenarios compared to full-tissue analysis. For example, GraphST’s performance gain increases from 8.3% on full tissue to 27.3% on subregions. This suggests that when spatial context is limited, LLM-derived gene functional knowledge helps anchor the identity of cell clusters, effectively compensating for the lack of local cell-cell interaction information. Based on these results, we recommend SpaLLM as a critical enhancement for experiments involving small biopsies or sparse cell populations where traditional methods often fail to recover clear domain boundaries.

**TABLE 7:** Comparative ARI analysis on osmFISH subregions (1,000 cells) versus full tissue.

Method	Subregion (1,000 cells)	Full tissue (4,839 cells)
SpaceFlow + SpaLLM (vs. base)	**0.41** vs. 0.35 (+17.1%)	**0.49** vs. 0.46 (+6.5%)
STAGATE + SpaLLM (vs. base)	**0.38** vs. 0.31 (+22.5%)	**0.43** vs. 0.39 (+10.2%)
GraphST + SpaLLM (vs. base)	**0.42** vs. 0.33 (+27.3%)	**0.52** vs. 0.48 (+8.3%)
stCluster + SpaLLM (vs. base)	**0.40** vs. 0.34 (+17.6%)	**0.47** vs. 0.44 (+6.8%)

Bold values indicate the best performance (highest ARI) for each dataset and quality level.

## 5 Conclusion and discussion

We presented SpaLLM, a general framework that integrates large language model embeddings of gene functional descriptions into spatial transcriptomics analysis. By leveraging pre-computed GenePT features and combining them with expression data through weighted matrix integration, SpaLLM consistently improves spatial domain identification across varying data quality conditions.

Our comprehensive evaluation on 12 sequencing-based DLPFC datasets and an independent imaging-based osmFISH dataset demonstrates substantial improvements in clustering accuracy. The gains range from 4% to 8% in high-quality data to remarkable 200%–400% improvements in severely degraded scenarios. The modular design enables seamless integration with existing spatial analysis methods including SpaceFlow, STAGATE, GraphST, and stCluster, making SpaLLM broadly applicable to diverse research scenarios regardless of the underlying experimental modality.

The success of SpaLLM demonstrates several key advantages: incorporating gene functional knowledge leads to more biologically meaningful clustering results, as evidenced by consistent improvements across all tested methods. Detailed ablation studies confirm that our weighted fusion strategy outperforms simple concatenation, and while newer models like *text-embedding-3-large* provide superior stability in extreme sparsity, *text-embedding-ada-002* remains a highly efficient default for routine analysis. Functional features provide stable signals even when expression data is sparse or degraded, with the most dramatic improvements observed in Q3 and Q4 quality scenarios. Furthermore, our subregion analysis reveals that SpaLLM is particularly transformative for small-scale tissue samples (e.g., 1,000 cells), where the relative improvement in ARI reaches up to 27.3%, effectively compensating for limited spatial context.

While SpaLLM shows consistent effectiveness, its current implementation depends on the accuracy of large language model embeddings for capturing gene functional relationships. However, with the rapid advancement of language model architectures and the continuous expansion of biological knowledge databases, we anticipate that this limitation will be progressively overcome, leading to even more precise functional representations that better capture the complexity of biological systems.

This work establishes a foundation for knowledge-guided spatial omics analysis and demonstrates the potential for large language models to enhance biological discovery through the systematic integration of functional knowledge. The consistent improvements across diverse datasets, varying tissue sizes, and methods suggest that functional knowledge integration represents a promising paradigm for advancing spatial transcriptomics analysis.

## Data Availability

The original contributions presented in the study are included in the article/supplementary material, further inquiries can be directed to the corresponding author.
